# Coordination Polymer: Synthesis, Spectral Characterization and Thermal Behaviour of Starch-Urea Based Biodegradable Polymer and Its Polymer Metal Complexes

**DOI:** 10.1155/2010/848130

**Published:** 2010-04-19

**Authors:** Ashraf Malik, Shadma Parveen, Tansir Ahamad, Saad M. Alshehri, Prabal Kumar Singh, Nahid Nishat

**Affiliations:** ^1^Materials Research laboratory, Department of Chemistry, Jamia Millia Islamia, New Delhi 110025, India; ^2^Department of Chemistry, King Saud University, Riyadh 1145, Saudi Arabia

## Abstract

A starch-urea-based biodegradable coordination polymer modified by transition metal Mn(II), Co(II), Ni(II), Cu(II), and Zn(II) was prepared by polycondensation of starch and urea. All the synthesized polymeric compounds were characterized by Fourier transform-infrared spectroscopy (FT-IR), ^1^H-NMR spectroscopy, ^13^C-NMR spectroscopy, UV-visible spectra, magnetic moment measurements, differential scanning calorimeter (DSC), and thermogravimetric analysis (TGA). The results of electronic spectra and magnetic moment measurements indicate that Mn(II), Co(II), and Ni(II) complexes show octahedral geometry, while Cu(II) and Zn(II) complexes show square planar and tetrahedral geometry, respectively. The thermogravimetric analysis revealed that all the polymeric metal complexes are more thermally stable than the parental ligand. In addition, biodegradable studies of all the polymeric compounds were also carried out through ASTM standards of biodegradable polymers by CO_2_ evolution method.

## 1. Introduction

Starch-based coordination polymers are known to be completely degradable in soil and water and can promote the degradation of nonbiodegradable material when blended or modified. Starch is one of the main natural polymers studied for the production of biodegradable materials [1]. Starch is a promising raw material because of its annual availability from many plants, its rather excessive production with regard to current need [2]. Because of environmental pollution problems caused by using synthetic polymers based on petrochemicals [3], the development of environment friendly polymeric material has attracted excessive interest [4]. A huge number of biodegradable polymers have been synthesized chemically or by microorganism and plants [5]. Depending on the origin, different categories of biodegradable polymers have been proposed. To list a few, there are agropolymers, such as starch or cellulose from agroresources, polymers obtained by microbial production, for example, polyhydroxyalkanoates, and chemically synthesized polymers from monomers derived from agroresources (e.g., polylactic acid). Chemically synthesized polymers from monomers obtained commercially by chemical synthesis. Among these, starch is potentially useful material for biodegradable material because of its natural abundance and low cost [6, 7]. Starch is the major carbohydrate in plant, tubes, and seed endosperm, where it is found as granule. Each granule contains several million amylopectin molecules accompanied by a much number of smaller amylase molecules. However, starch-based-materials have some drawbacks [8] including limited long-term stability caused by water absorption, aging caused by retrogradation, poor mechanical properties, and bad processability. To overcome these limitations, biodegradable polymers have been synthesized and modified. Our purpose of work was the modification of starch with urea for improving the properties and with some transition metals for enhancement in the characteristics and for synthesizing the coordination polymer. The applications of biodegradable polymers have been discussed on three major areas, namely, medical, agricultural and consumer goods packaging. Some of these have resulted in commercial products because of their specialized nature and greater unit value. Medical device applications have developed faster than the other two and have been used as surgical implants in vascular and orthopedic surgery as implantable matrices for the controlled long-term release of drugs inside the body as absorbable surgical implants and for use in the eyes; some other applications are bone fixation devices and vascular grafts, adhesion prevention, artificial skin, drug delivery system, agricultural mulches controlled release of agricultural chemicals, agricultural planting containers, and packaging [9–12].

## 2. Experimental

### 2.1. Materials

Starch, urea, ethanol MERCK (Mumbai), and sodium hydroxide were used without further purification. Solvents such as acetone, DMF, DMSO, and (s.d fine chemicals) methanol were purified by standard procedure before use. Manganese (II) acetate tetrahydrate [Mn(CH_3_COO)_2_ · 4H_2_O], copper (II) acetate monohydrate [Cu(CH_3_COO)_2_ · H_2_O], nickel (II) acetate tetrahydrate [Ni(CH_3_COO)_2_ · 4H_2_O], cobalt (II) acetate tetrahydrate [Co(CH_3_COO)_2_ · 4H_2_O], and zinc (II) acetate dihydrate [Zn(CH_3_COO)_2_ · 2H_2_O] were used without further purification. All the microorganisms were provided by C.S.A. Agricultural University, Kanpur.

### 2.2. Synthesis

#### 2.2.1. Synthesis of Polymeric Resin

**Scheme 1:**
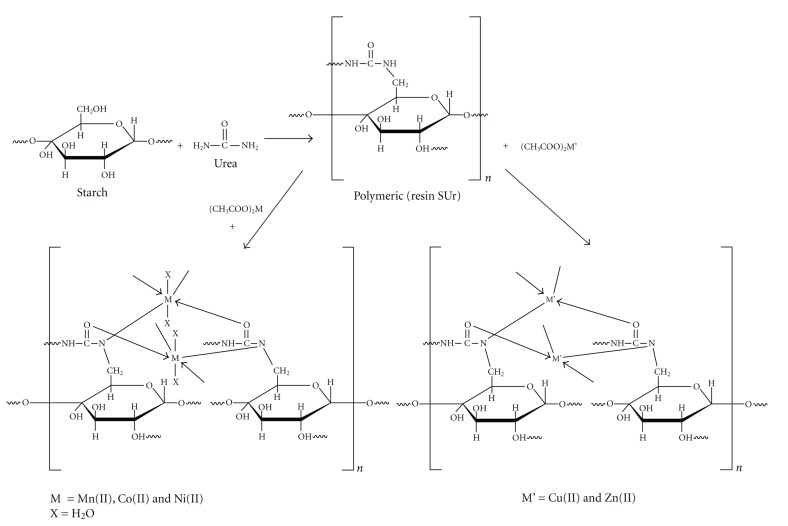


The polymeric resin was synthesized by polycondensation of urea and starch in alkaline medium in 1 : 1 molar ratio according to Scheme 1 [13, 14]. In a 250 mL three-necked round-bottomed flask, equipped with a stirrer and a condenser, 0.60 gm (0.01 mol) of urea and 1.62 gm (0.01 mol) of starch poured with 110 mL deionized water were placed, and then it was stirred with high speed (>1000 r/min) in a constant temperature water bath at 95°C for 3 h. The pH was adjusted to 8 with NaOH. The reaction was monitored by thin layer chromatography (TLC) using ethanol as an eluent. The resulting colorless viscous product was washed with ethanol and acetone and dried in a vacuum oven under reduced pressure at 50°C for 10 h. The white powder of starch-based polymer modified by urea (poly-SUr) was obtained in 70% yield. The synthesized product was found to be soluble in distilled water and DMSO and insoluble in some common organic solvents.

#### 2.2.2. Synthesis of Metal Complexes

Metal complexes of poly-SUr were prepared by using molar ratio (1 : 1) of poly-SUr and metal salts. A typical procedure for the preparation of the Cu(II) complex is carried out as 2.22 gm (0.01 mol) of poly-SUr dissolved in a minimum quantity (~25 mL) of hot DMSO and 1.99 gm (0.01 mol) of Cu(II) salt was dissolved in DMSO (~20 mL) separately. Both solutions were filtered and mixed in hot condition with constant stirring. Then the reaction mixture was stirred at 60°C for 4 h. A dark green colored product was obtained which was reprecipitated in distilled water. Finally the product was filtered and washed with alcohol, acetone and dried in a vacuum desiccator on calcium chloride, yield 75%.

 A Similar procedure was adopted for the synthesis of the other metal complexes such as poly-SUr-Mn (II), poly-SUr-Co(II, poly-SUr-Ni(II)), and poly-SUr-Zn(II); their yields were between 73%–78%, and the obtained product was found to be soluble in dimethylsulfoxide-d6(DMSO- d6) and insoluble in some common organic solvents and distilled water.

### 2.3. Measurements

The infrared (IR) spectra were recorded on a Perkin-Elmer infrared spectrometer model 621 by using KBr pellets. The ^1^H-NMR spectra were recorded on a JOEL-FX-100 FT NMR instrument in dimethylsulfoxide (DMSO) solution and tetramethylsilane (TMS) as an internal standard. The elemental analysis of carbon, hydrogen, and nitrogen was carried out on a Perkin-Elmer model-2400 elemental analyzer (CDRI Lucknow). The percentage of metals was determined by complexometric titration against EDTA after decomposing with concentrated nitric acid (HNO_3_). The solubility of polymeric ligand and its metal polychelates were checked at room temperature in different solvents. The thermal stability of polymer and its metal polychelates have been evaluated for recording thermograms by TA analyzer 2000 at a heating rate of 20°C per minute under Nitrogen atmosphere. The Tg and Tm of the synthesized polymeric resin (SUr) and its metal complex have been evaluated by PYRES DIAMOND DSC instrument. The electronic spectra of the metal complexes were recorded on a Perkin-Elmer Lambda-EZ 201, and magnetic susceptibility measurements were done with vibrating sample magnetometer. The biodegradable testing was carried out through CO_2_ evolution method in the laboratory itself.

## 3. Results and Discussions

The polymeric resin (poly-SUr) was prepared by the polycondensation process in the molar ratio of 1 : 1 in alkaline medium, according to Scheme 1, and metal complexes were prepared by the reaction of poly-SUr with metal acetate in 1 : 1 molar ratio. All the products were obtained in good yields. The polymeric resin was found to be soluble in water and DMSO while all the metal complexes were soluble in DMSO only and insoluble in common organic solvents like methanol, ethanol, THF, DMF, CHCl_3_, CCl_4_, and so forth. The elemental and spectral analysis provide good evidence that the compounds are polymeric and these data are also in agreement with the molecular structure given in Scheme 1; the results of elemental analysis and yields of the synthesized compounds are given in Table 1.

### 3.1. FTIR Spectra

The important IR bands and their assignments of polymeric resin (poly-SUr) and its metal complexes are listed in Table 2. In the IR spectra of poly-SUr, bands that appeared in the region 3402 cm^−1^ for *ν*OH. The vibrational bands appeared at 1250 cm^−1^ and 1080 cm^−1^ are assigned to the *ν*C-N and *ν*C-O, respectively. The bands were observed at 2936-2850 cm^−1^ for CH_2_ asymmetric and symmetric stretching vibrations. For *ν*O=C-NH (amide carbonyl linkage), vibrational bands appeared at 1778 cm^−1^ and N-H bending appeared at 1654 cm^−1^. In the case of metal complexes, vibrational bands appeared for O=C-NH (amide carbonyl linkage) shifted to the lower frequencies and indicating the coordination of amide nitrogen to metal ion, and this can be explained by the donation of electrons from nitrogen to metal atom. The *ν*C-O also registered a significant shift to lower frequency indicating the participation of metal through the phenolic oxygen. The *ν*O-H also registered lowering in frequency [15]. In all the polymer metal complexes, some additional bands also appeared in the regions of 610–603 cm^−1^ and 552–548 cm^−1^, which support the coordination of metal through oxygen and nitrogen [16, 17].

### 3.2. ^1^H-NMR Spectra

The ^1^H-NMR bands ranges of polymeric resin and its metal complex with Zn (II) are given in Table 3. The resin showed a signal at 8.394 ppm for O=C-NH protons of amide carbonyl group. The bands appeared at 5.079–5.003, 4.718, 3.699, and 5.557 ppm due to pyranose ring of starch. Bands appeared at 3.45–3.32 ppm due to OH protons [18]. In the spectrum of poly-SUr-Zn (II) complex, the bands for O=C-NH shifted from their original position at 7.940 ppm, which suggested the involvement of metal ions in coordination [18].

### 3.3. ^13^C-NMR Spectra

The ^13^C-NMR band ranges of polymeric resin (poly-SUr) and its metal complex with Zn(II) are given in Table 4. The ^13^C-NMR bands of poly-SUr showed resonance band at 159.42 ppm due to the carbonyl carbon of amide. The pyranose carbons of starch molecules in the polymeric resin showed bands in the region 98.14, 69.89, 77.20 and 55.67 ppm, respectively [19, 20]. In case of metal complex bands showed shifting in comparison with the corresponding bands of polymeric resin as 155.76, 78.88, 64.53, 71.63, and 54.12 ppm, which also support the participation of metal ions.

### 3.4. Electronic Spectra and Magnetic Susceptibility Measurement

The electronic spectra of metal complexes were recorded in DMSO. The electronic spectral bands and their magnetic moments are depicted in Table 5. The magnetic moment of the Mn (II) complex was 5.69 B.M., Which suggested the presence of five unpaired electrons. The electronic spectrum of polymer complex of Mn(II) exhibited three bands at 18870 cm^−1^, 22980 cm^−1^, and 25330 cm^−1^, which may reasonably correspond to ^4^T_1g_(G) *←* ^6^A_1g_(F), ^4^T_2g_(G) *←* ^6^A_2g_(F), and ^4^A_1g_(G) *←* ^6^A_1g_(F) transitions, respectively, which indicates an octahedral environment around the Mn(II) ion [21], and its ligand field parameters 10Dq, B, *β* and *β* ^0^ values are 7960, 625, 0.68 and 32%. The polymer complex of Co(II) has a magnetic moment of 4.10 B.M. corresponding to four unpaired electrons and showed three bands at 9800 cm^−1^, 14080 cm^−1^ and 20400 cm^−1^ which were assigned to ^4^T_2g_(F) *←* ^4^T_1g_(F), ^4^A_2g_(F) *←* ^4^T_1g_(F), and ^4^T_1g_(P) *←* ^4^T_1g_(F) transitions, respectively, which indicates an octahedral environment around the Co(II) ion [22, 23], and its ligand field parameters 10Dq, B, *β* and *β* ^0^ values are 6193 cm^−1^, 729 cm^−1^, 0.75 and 25%, respectively. The octahedral polymer complex of Ni(II) was expected to be paramagnetic owing to two unpaired d-electrons, and the experimental magnetic moment was found to be 2.77 B.M. The electronic spectra showed three bands at 8355 cm^−1^, 12155 cm^−1^, and 23809 cm^−1^which were assigned to ^3^T_2g_(F) *←* ^3^A_2g_(F), ^3^T_1g_(F) *←* ^3^A_2g_(F), and ^3^T_1g_(P) *←* ^3^A_2g_(F) transition, respectively, which is in the favor of octahedral geometry for polymer complex of Ni(II) [24], and its ligand field parameters 10Dq, B, *β* and *β* ^0^ values are 5952 cm^−1^, 744 cm^−1^, 0.69, and 31%, respectively. The above discussion very strongly indicates an octahedral geometry around the central metal ion in all metal complexes. It accounts for the occupation of two coordinating sites by H_2_O out of six in making the octahedral environment.

 In another study the electronic spectra of the SUr-Cu(II) exhibited two bands, at 15380 cm^−1^ and 25000 cm^−1^ due to ^2^A_1g_ *←* ^2^B_1g_(F) and charge transfer spectra, respectively, which indicate square planar geometry [25]. In the present study the magnetic moment value of SUr-Cu(II) was found to be 1.9 B.M., which is in accordance with square planar geometry. The SUr-Zn(II) is diamagnetic and showed tetrahedral geometry.

### 3.5. Thermogravimetric Analysis

**Figure 1:**
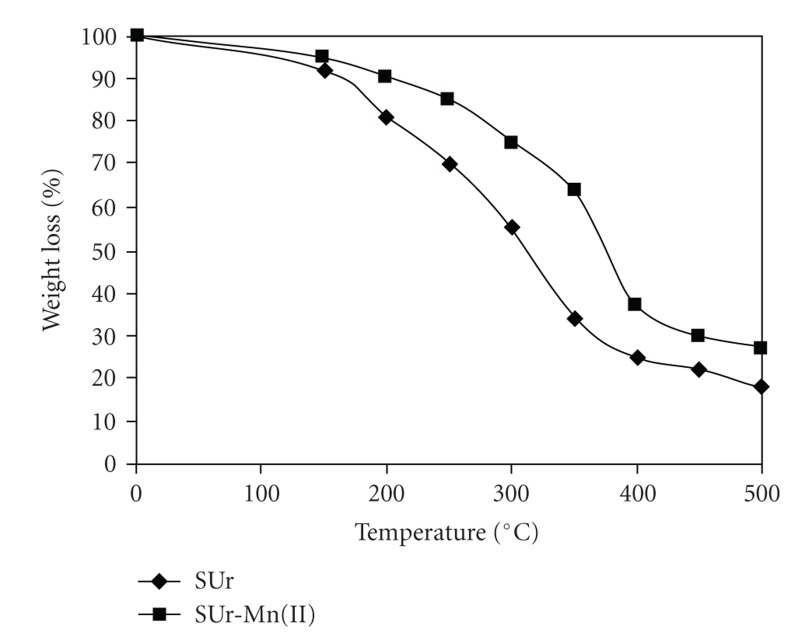
TGA curve of SUr and SUr-Mn(II).

The thermal decomposition of polymeric resin and its polymer metal complex [SUr-Mn(II)] were studied by the thermogravimetric method. The thermogravimetric curves of polymeric resin and its polymer metal complex are depicted in Figure 1 and the thermal analytical data are listed in Table 6. The polymeric resin started to decompose with weight loss from 7%–8% at 150°C because the boiling point of the plasticizer outclassed 150°C was mainly ascribed to water loss, and the mass loss from 150°C to the onset temperature was related to the volatilization of both water and plasticizer. The decomposition of polymeric resin was slow in the initial stage, but above 200°C, weight loss became fast. The thermal data indicate that the thermal stability of the poly-SUrs upto 250°C; however, its metal complexes decrease the decomposition and reduced mass loss at the onset temperature. The volatility of the polymer metal complex was reduced because of the formation of coordination bond with the polymeric resin. Polymer metal complex started to decompose with weight loss 4%-5% at 150°C and the decomposition rate of metal complexes was very slow upto 275°C, but above 275°C, weight loss became fast. This result revealed that metal complex [SUr-Mn(II)] show better heat resistant characteristics than these of the polymeric resin due to the coordination of metal ions.

### 3.6. Differential Scanning Calorimetry (DSC)

**Figure 2:**
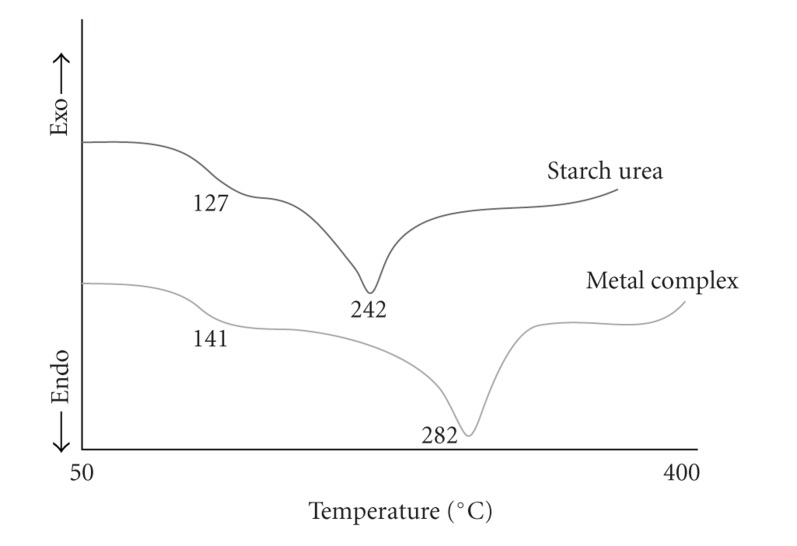
DSC curves of starch urea and its metal complex.

The DSC measurement served to determine the glass transition temperature Tg and decomposition behavior, and for this, the calorimetric curves of the polymeric resin and its polymer metal complexes are reported in Figure 2. The decomposition temperature scattered typical range is of 242°C for polymeric resin and the Tg scattered is in the range of 127°C for polymeric resin; this evolution could be ascribed due to the interaction between the starch and urea. The strong hydrogen bond formed between starch and urea, which decreased starch chain mobility and consequently increased the matrix glass transition temperature, but in the case of polymer metal complexes, the decomposition temperature and Tg increased. The decomposition temperature scattered typical range for polymer complex of Co(II) is 282°C and the Tg scattered is in the range of 141°C. This is the evidence that the thermal stability is enhanced by the incorporation of metal into the organic backbone. The glass transition temperature of these polymers increased by metal incorporation, and lower Tg and Tm are related to amount of starch and urea in the initial composition. This is the first evidence that starch participates in the chemical reaction and is becoming part of macromolecular structure. In addition, the Tg variation of metal incorporated polymer depends on the amount of metal ions and also the cross-linking effect of polymer between two chains of starch and strong hydrogen bonding which affects on chain mobility of starch modified in different ways.

### 3.7. Biodegradable Testing

It is a laboratory respirometric method that uses compost pile inocula given in Table 7(a) as a biotic function, [26] but the user may use other microbial environmental interest, with certain typical polymers that are readily biodegradable (such as aliphatic polyester or polyamides) and with biopolymer materials (paper, cotton cloth, cellulose, cellophane); it is convenient to use especially designed flask for this purpose (Anthony L. Andrady (1998) by Chapman and Hall London).

### 3.8. Biotic Medium

The test method might be carried out in water or soil but latter one is discussed here. Although in both cases a suitable volume of activated sludge to obtain complete mineralization of the sample in about a month is used as inoculums, about 10 cm^3^ per liter is recommended for aqueous system and we have used 5 cm^3^ per 50 g with cold medium successfully. The inoculums must be used the same day as collected and kept aerated until used. Also sufficient urea and potassium hydrogen phosphate (0.1 and 0.05 % of weight of polymer substrate) are added to the medium to fortify it and to promote further microbial growth.

**Figure 3:**
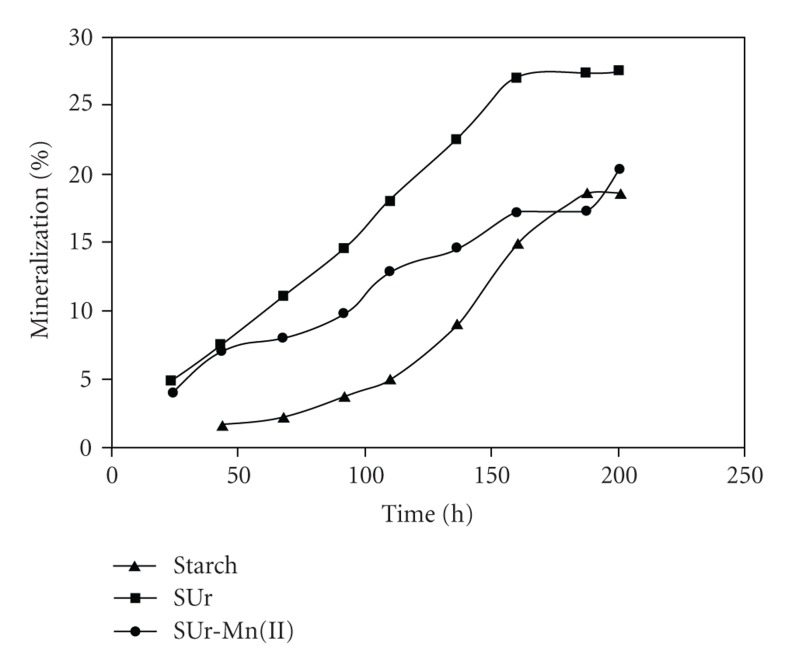
%CO_2_ mineralization of starch, SUr and SUr-Mn(II).

 The biodegradation rates of starch, polymeric resin, and polymer metal complexes for a comparative study are shown in Figure 3 and predicted in Table 7(b) and 7(c), respectively. From these figure and Table, it can be seen that the biodegradation rates of polymer metal complexes were lower than those of starch polymer after 187 hrs, and that the biodegradation rates of polymeric resin and starch polymer were in good coherence after 187 hrs and 200 hrs, respectively, that would be because the starch and urea support to the microorganism growth and degradation behavior.

## 4. Conclusion

Newly developed polymeric resin and its metal complexes were prepared in good yield and characterized by various instrumental techniques. The polymeric resin was soluble in water and DMSO and insoluble in benzene, toluene, and methanol, whereas all the metal complexes were soluble in DMSO and insoluble in water and common organic solvents. It has been observed that the incorporation of metal ion in the polymeric backbone enhances the thermal properties as well as reduced biodegradability, because of the degradable nature of the prepared polymeric resin, which is coordinated to metal ions, and they may be used in various applications of biomedicines and plastic technology. The intermolecular hydrogen bonding of the starch was decreased due to the addition of urea and the thermal behavior of the entire polymer increased by the incorporation of metal salts into the polymeric backbone.

## Figures and Tables

**Table 1:** Elemental analysis and yields of the synthesized polymeric compounds.

Compounds	Yield	d.p.	Elemental analysis			
	(%)	(°C)	% C	% H	% N	% M
SUr	70	242	38.183	5.43	12.78	—
			37.10	4.64	12.85	
SUr-Mn(II)	74	282	27.44	5.01	9.10	18.11
			26.39	4.02	9.45	18.22
SUr-Co(II)	73	292	26.84	4.50	8.99	18.81
			26.57	4.11	8.37	18.65
SUr-Ni(II)	76	299	26.68	4.51	9.10	18.75
			26.10	4.36	9.97	18.10
Sur-Cu(II)	75	295	29.83	3.58	10.01	22.54
			30.10	4.31	10.97	22.12
Sur-Zn(II)	78	286	29.63	3.55	9.94	23.04
			30.70	3.20	9.88	23.33

**Table 2:** IR bands of polymeric resin (SUr) and its polymer metal complexes.

Compounds	O-H	*υ*C-O	*υ*C-N	CH_2_ asym-sym	O=C-NH	*δ*N-H	M-O	M-N
SUr	3402	1080	1250	2936-2850	1778	1654	—	—
SUr-Ni(II)	3365	1050	1210	2936-2850	1658	1610	610	550
SUr-Mn(II)	3362	1051	1211	2936-2850	1657	1612	609	551
SUr-Zn(II)	3359	1049	1213	2936-2850	1660	1614	607	552
SUr-Cu(II)	3367	1052	1216	2936-2850	1659	1613	605	548
SUr-Co(II)	3364	1053	1215	2936-2850	1660	1615	603	549

**Table 3:** Number of protons in different environment of SUr and its polymer metal complexes.

Polymeric resin		Polymer metal complex of Zn(II)	
Functional groups	Peaks	Functional groups	Peaks
O=C-NH	8.394(h)	O=C-NH	7.94
protons of pyranose rings	5.079(a)	protons of pyranose rings	5.418(e)
	5.003(d)		5.000(d)
	4.718(b)		4.510(b)
	3.6999©		3.330©
	5.557(e)	O-H protons	3.415(f)
	3.45(f)		2.666(g)
	3.32(g)		

**Table 4:** Number of carbon atoms in different environment of SUr and its polymer metal complexes.

Polymeric resin		Polymer metal complexes	
Functional groups	Peaks	Functional groups	Peaks
O=C-NH	159.42(a)	O=C-NH	155.76
Pyranose carbons	98.14(b)	pyranose carbon	78.88(b)
	69.89(c,d,f)		64.53(c,d,f)
	77.20(e)		71.63(e)
	55.67(g)		54.12(g)

**Table 5:** Electronic spectral bands and magnetic moment measurements of polymer metal complexes.

Complexes	Magnetic moment (B.M.)	Bands cm^−1^	Transitions	Geometry	10Dq	B	*β* ^0^	*β*%
SUr-Mn(II)	5.69	25330	^4^A_1g_(G)*←* ^6^A_1g_ (F)					
		22980	^4^T_2g_(G)*←* ^6^A_1g_(F)	Octahedral	7960	625	0.65	35
		18870	^4^T_1g_(G)*←* ^6^A_1g_(F)					
SUr-Co(II)	4.10	20400	^4^T_1g_(F)*←* ^4^T_1g_(F)					
		14080	^4^A_2g_(F)*←* ^4^T_1g_(F)	Octahedral	6193	729	0.75	25
		9800	^4^T_2g_(F)*←* ^4^T_1g_(F)					
SUr-Ni(II)	2.77	23809	^3^T_1g_(P)*←* ^3^A_2g_(F)					
		12155	^3^T_1g_(F)*←* ^3^A_2g_(F)	Octahedral	5952	744	0.69	31
		8335	^3^T_2g_(F)*←* ^3^A_2g_(F)					
SUr-Cu(II)	1.90	25000	Charge transfer spectra	Square planar	—	—	—	—
		15380	^2^A_1g_ *←* ^2^B_1g_ (F)	—	—	—	—	—
SUr-Zn(II)		—	—	Tetrahedral	—	—	—	—

**Table 6:** Thermal behaviors of SUr and its polymer metal complex of Mn(II).

Polymeric resin (SUr)		SUr-Mn(II)	
Temperature (°C)	Weight loss (%)	Temperature (°C)	Weight loss (%)
150	8	150	5
200	11	200	5
250	11	250	5
300	15	300	10
350	21	350	11
400	9	400	27
450	3	450	7
500	4	500	3

**Table:** (a) ASTM standards of biodegradable polymeric compounds.

Environment	ASTM	Microorganism
Single species	D5247-92, G-21, G-22	Species of bacteria and fungi specified
Sewage sludge	D5209-91, D5271-92	Aerobic-activated sludge organisms
Marine environment	D5437-93	Marine algae and invertebrates
Compost pile	D5338-93	Thermophillic microbes in compost
Anaerobic environment	D5210-92	Anaerobic micros from activated sludge

**Table:** (b) % CO_2_ mineralization of starch, SUr, and its polymer metal complex.

Time (Hrs)	CO_2_ Mineralization %		
	Starch	Polymeric resin	Polymer metal complex
24	—	4.8	4
44	1.6	7.5	7.1
68	2.2	11	8
92	3.7	14.5	9.7
110	5	18	12.8
136	9	22.5	14.5
160	15	27	17.2
187	18.5	27.4	17.3
200	18.6	27.5	20.4

**Table:** (c) Biodegradability of starch, SUr and SUr-Mn(II).

Material	Biodegradability rate constant (k)	Total Wt. loss (gm)	Medium
Starch	0.001297	0.0108	Biotic
SUr	0.00196	0.01527	Biotic
SUr-Mn(II)	0.001163	0.00906	Biotic
